# High-Order Harmonic Generation in Au Nanoparticle-Contained Plasmas

**DOI:** 10.3390/nano10020234

**Published:** 2020-01-29

**Authors:** Mottamchetty Venkatesh, Rashid A. Ganeev, Dmitry S. Ivanov, Ganjaboy S. Boltaev, Vyacheslav V. Kim, Jingguang Liang, Andrey A. Samokhvalov, Andrei V. Kabashin, Sergey M. Klimentov, Martin E. Garcia, Chunlei Guo

**Affiliations:** 1 The Guo Photonics Laboratory, State Key Laboratory of Applied Optics, Changchun Institute of Optics, Fine Mechanics and Physics, Chinese Academy of Sciences, Changchun 130033, China; venkatesh@ciomp.ac.cn (M.V.); gboltaev@aus.edu (G.S.B.); vkim@aus.edu (V.V.K.); lijigu123@163.com (J.L.); 2 Department of Physics, American University of Sharjah, 26666 Sharjah, UAE; 3 Faculty of Physics, Voronezh State University, 394006 Voronezh, Russia; 4 Institute of Physics and Center for Interdisciplinary Nanostructure Science and Technology (CINSaT), University of Kassel, 34125 Kassel, Germany; m.garcia@uni-kassel.de; 5 National Research Nuclear University MEPhI (Moscow Engineering Physics Institute), 115409 Moscow, Russia; kabashin@lp3.univ-mrs.fr (A.V.K.); kliment-61@mail.ru (S.M.K.); 6 P. N. Lebedev Physical Institute of Russian Acad. Sci., 119991 Moscow, Russia; 7 Department of Laser Photonics and optoelectronics, ITMO University, 197101 St. Petersburg, Russia; samokhvalov.itmo@gmail.com; 8 Tashkent Institute of Irrigation and Agricultural Mechanization Engineers, Tashkent 100000, Uzbekistan; 9 Aix Marseille Univ, CNRS, LP3, Campus de Luminy, Case 917, 13288 Marseille, France; 10 The Institute of Optics, University of Rochester, Rochester, NY 14627, USA

**Keywords:** ultrashort pulses, laser plasma, nanoparticles, high-order harmonics, molecular dynamics, simulation, laser-matter interactions

## Abstract

Gold nanoparticles (NPs) have a wide range of applications in various fields. Here, we present high-order nonlinear optical studies of the plasmas produced from ablation of Au bulk targets and Au NP films deposited on paper and glass substrates. Experimentally, we analyze high-order harmonic generation (HHG) from gold NPs-containing plasmas. The HHG is produced by 35-fs pulses at 800 and 400 nm, while the plasmas are produced by femtosecond (35 fs, 800 nm), picosecond (200 ps, 800 nm), and nanosecond (5 ns, 1064 nm) pulses, respectively. High-order harmonics produced from ablated Au NPs on paper were 40 times stronger than the HHG from that ablated from the Au bulk targets. Through molecular dynamic simulations, we investigate the formation of gold NPs during laser ablation of a metal surface under different conditions.

## 1. Introduction

Rare gases and laser-produced plasmas (LPP) from solid targets have been used as the nonlinear media for high-order harmonic generation (HHG) in extreme ultraviolet (XUV) range [1,2]. Currently, the best achieved conversion efficiency of harmonics is in the range of 10^−6^–10^−5^ [3,4]. The HHG efficiency is limited by the harmonic absorption in the generating media and the phase mismatch between the driving field and the harmonics.

The laser plasma plumes are the suitable media for generation of low- and high-order harmonics. The application of LPP allowed studies of different processes, such as the resonance enhancement of single harmonic, quasi-phase matching in multi-jet plasmas, and nanoparticle-enhanced harmonics [5,6,7,8,9,10,11,12,13,14,15,16]. Earlier studies have shown that harmonics emitted from LPPs are more intense with regard to gaseous media [17]. The plasmas containing clusters, nanoparticles (NPs), and large molecular systems enhance the harmonic yield with regard to atomic and ionic structures. Recently, nanostructured and ion-implanted semiconductor targets paved a path to produce strong high-order harmonics as compared to un-patterned samples [18,19].

There is an urgent need to search for methods to generate intense harmonics and attosecond pulses. For these purposes, gas clusters and plasma produced from nanoparticle targets can be used to increase the intensity of harmonics. The motivation of these studies is related with the comparison of the high-order nonlinear optical response of the nanoparticles appearing from the targets during ablation of NP-contained samples and bulk Au samples. The dynamics of plasma formation in the former case can be considered as follows. The material directly surrounding the NPs is a polymer (epoxy glue), which has a considerably lower ablation threshold than the metallic materials. The NPs absorb the laser pulse energy and pass the thermal energy to the surrounding media. Therefore, the NPs-carrying polymer begins to ablate at relatively low intensities, resulting in the lower laser fluence required for the preparation of the appropriate nonlinear medium for the HHG. This feature allowed for easier creation of the optimum plasma conditions, which resulted in a better HHG conversion efficiency from the Au NPs-containing plume compared to the plasma from the bulk target.

The production of clusters in the laser plasma during laser ablation of various targets has a high probability, while their presence and concentration in the plasma volume, where the frequency conversion occurs, is yet to be confirmed directly. Analysis of post-ablation conditions of the deposited debris can provide information on the nature of those species, despite the differences between the composition of the plasma formed at its early stages and the deposited material (due to the influence of aggregation on the substrate). Further evidence of the cluster contribution to the enhancement of the harmonic generation comes from investigations of intense laser ablation of a target, which would give the assumptions regarding the participation of in situ generated nanoparticles.

Gold nanomaterials have potential applications because its surface plasmon resonance affects the optical properties of materials. Here, we study how harmonics obtained from Au NPs are affected by using the active substrate, which itself allows the generation of harmonics. In order to support the experimental measurements and to advance in understanding of the fundamental physical processes underlying the laser-induced formation of Au NPs we perform a series of computer simulations of laser ablation of thick gold target at different irradiation parameters. The used model is capable of addressing the kinetics of strongly non-equilibrium laser-induced phase transition processes at atomic level with Molecular Dynamics (MD) approach.

In this paper, the systematic experimental and numerical studies of the NPs formation during laser ablation and the HHG from Au NPs, Au bulk, paper and Au NPs deposited on paper and glass substrates were carried out. These studies also allow understanding the effects of single-color pump (SCP) and two-color pump (TCP) of plasma, laser chirping, laser pulse duration on the harmonic conversion efficiency from different ablated targets, and the choice of irradiation regimes for generation of NPs with predesigned optical and morphological properties.

## 2. Materials and Methods

### Experiment

**Figure 1:**
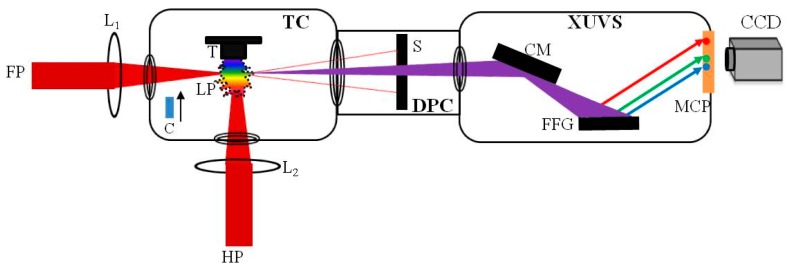
Experimental setup for high-order harmonic generation in LPPs. FP, converting femtosecond pulses, HP, heating pulses; L_1,2_, focusing lenses; TC, target chamber; T, target; C, BBO crystal; LP, laser plasma; S, slit; DPC, differential pump chamber; XUVS, extreme ultraviolet spectrometer; CM, cylindrical gold-coated mirror; FFG, flat field grating; MCP, micro-channel plate; CCD, CCD camera.

The experimental layout of HHG is shown in Figure 1. In total, 100 nm and 10 nm Au NPs (SkySpring Nanomaterials Inc., Houston, TX, USA, 99.95% purity) were glued on the glass substrate and paper, respectively. Notice that ablated pure glue did not allow generation of harmonics in LPP. The studied samples were ablated by nanosecond (1064 nm, 5 ns, 10 Hz; Q-Smart, Coherent, Palo Alto, CA, USA), picosecond (800 nm, 200 ps, 200 Hz) and femtosecond (800 nm, 30 fs, 200 Hz) pulses. Both femtosecond (fs) and picosecond (ps) laser pulses were obtained from the same laser (Spitfire Ace; Spectra Physics, Santa Clara, CA, USA) by separation of the part of uncompressed radiation (200 ps) before entering into the compressor stage. The delay between heating radiation (i.e., fs or ps pulses) and driving fs pulses (800 nm, 30 fs, 200 Hz) was varied in the range of 0–120 ns by using the optical delay line. The delay between heating nanosecond (ns) pulses and driving fs pulses was varied electronically between 0 and 10^5^ ns using a delay generator (DG535; Stanford Research Systems, Sunnyvale, CA, USA). LPP was created by heating pulses, while the driving pulses were focused inside the plasma using 500 mm focal length spherical lens from the orthogonal direction with regard to ablating pulse to generate harmonics. The diameter of focused driving femtosecond pulses was 64 µm. The energy (intensity) of driving pulses employed in our experiment was 0.5 mJ (4 × 10^14^ W cm^−2^). The HHG experiments using TCP of LPP were carried out using the 0.2-mm thick β-barium borate (BBO) crystal, which was kept inside the vacuum chamber on the path of 800 nm pulses to generate second harmonic (400 nm). The harmonic spectra were analyzed using a XUV spectrometer and collected by a CCD camera.

## 3. Results

### 3.1. Comparison of Harmonic Emission from Different Plasmas Containing Gold Nanoparticles

The low- and high order nonlinear response of gold NPs prepared by chemical method was analyzed in Ref. [20]. Particularly, the harmonics up to the 27th order (H_27_) were generated during ablation of thin Au NP-containing films. The difficulties encountered during application of these films were related with their small thickness (100 nm) leading to evaporation during a single shot, which required the constant movement of the destroyed film. In present study, we use the commercially available Au NP powder, which can be attached to the surface of different materials (glass, paper) to form the NP multilayer sample with rather large thickness (~1–2 mm). The application of such samples allowed the maintenance of relatively stable harmonic emission for a longer period of ablation. This amendment in handling the NP target, in turn, allowed better optimization of HHG and achieving the conditions for generation of higher-order harmonics (up to H_39_). Additionally, we analyzed HHG from the ablated bulk gold target. Our simulations demonstrated the appearance of Au NPs in LPP at these conditions, which can also enhance the harmonic yield from such plasma.

We analyzed HHG spectra using the 1 s integration time of CCD. The employed repetition rates of ns, fs and ps laser were 10, 200, and 200 Hz, respectively. Every single HHG spectrum was obtained from the fresh sample. The irradiation of the same spot of targets at high pulse repetition rate caused the crater formation and degradation of plasma thus decreasing the stability of harmonic yield. We firstly maintained the stable harmonics generation by moving the position of the focal spot of heating radiation along the horizontal axis of the targets. The movement was accomplished manually and was restricted by the length of the targets (5 mm). At any movement of plasma plume it was positioned within the confocal parameter (8 mm) of the focused driving pulses. We were also able to move the target along the vertical and horizontal axes using the computer-driven three-axis translating stage. However, the most advanced method was the application of the rotating target, which has earlier allowed stable harmonics generation at least for 10^6^ shots corresponding to ~20 min of instant irradiation of the target by 1 kHz class laser [21]. The rotating speed in the range of 5–300 rpm did not influence the stability of harmonics. The application of rotation target and the movement of the spot of heating beam along the height of this target being dragged up and down would allow further improvement of the stability of harmonic yield.

**Figure 2:**
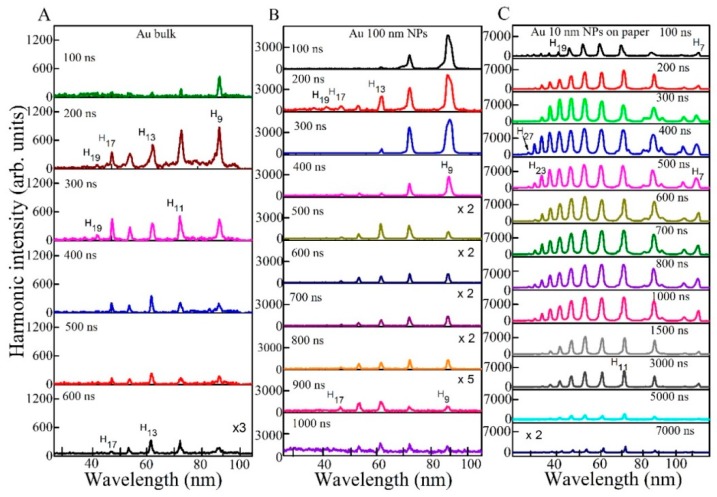
Harmonics spectra from the Au plasmas produced on three targets at different delays between the heating and driving pulses. (**A**) Bulk Au, (**B**) Au 100 nm NPs, (**C**) Au 10 nm NPs glued on paper.

Figure 2 shows the harmonics in the spectral range of 20–130 nm generated from the plasmas produced on the surface of different targets (Au bulk target, Au 100 nm nanoparticles, and Au 10 nm nanoparticles glued on paper). In total, 5 ns, 1064 nm, 10 Hz and 35 fs, 800 nm, 200 Hz laser pulses were used as the heating and driving pulses. These HHG spectra show the variations for different delays between the driving and heating pulses (from 100 to 7000 ns) at heating nanosecond and femtosecond pulse energies of 10 and 0.5 mJ, respectively. At smaller delays (<20 ns), the concentration of particles (neutral atoms, molecules, single charged ions and NPs) was insufficient for HHG because the whole cloud of ablated particles possessing velocities of ~2 × 10^4^ m s^−1^ cannot reach the spatial region of the driving beam, which propagated at the distance of 0.5 mm above the targets surfaces. At larger delays (≥100 ns), the concentration of the particles appearing on the path of driving pulse became sufficient for the generation of harmonics.

The optimal delays allowing generation of the maximal yield of harmonics were in the range of 150–400 ns for the ablated Au bulk, Au 100 nm NPs and Au 10 nm NPs on paper. At optimum delay, the harmonic range was extended up to H_19_ for Au bulk and Au 100 nm NPs, whereas it was H_29_ in the case of ablated Au 10 nm NPs on paper. An increase of the delay above the optimal values led to gradual decrease of HHG efficiency. The harmonics were observed up to 600, 1000 and 7000 ns delays between the driving and heating pulses for the ablated Au bulk, Au 100 nm NPs, and Au 10 nm NPs on paper, respectively.

**Figure 3:**
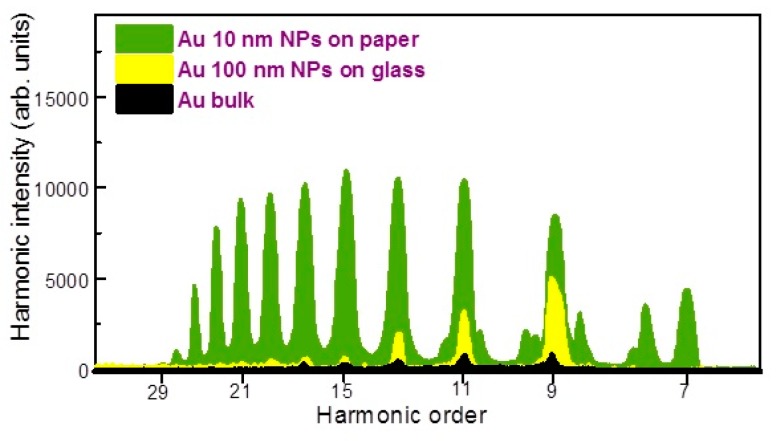
Comparative harmonic spectra from ablated bulk Au (at 200 ns delay), Au 100 nm NPs (at 200 ns delay), and Au 10 nm NPs on paper (at 350 ns delay).

Figure 3 shows the comparative spectra of harmonics produced from the plasmas generated on the Au bulk, Au 100 nm NPs deposited on glass, and Au 10 nm NPs on the paper at optimal delays between the heating and driving pulses. Some emission lines appeared in harmonic spectra and were attributed to the carbon presented in paper-containing target. The emission lines were determined using the NIST Atomic Spectra Database Lines [22].

At 200 ns delay, the harmonic intensity obtained from ablated Au 10 nm NPs on paper was approximately three and 14 times stronger with regard to Au NPs on glass and Au bulk, respectively. However, at 500 ns delay, the harmonic yield from ablated Au 10 nm NPs on paper was further enhanced up to five and 40 times compared to Au 100 nm NPs on glass and bulk Au, respectively. No harmonic was observed in the case of heating of the pure glass surfaces at similar fluencies of heating pulses.

The weak harmonics appearing from paper plasma were attributed to the presence of the carbon atoms and ions in LPP. Previously, the presence of CI and CII in carbon-containing plasma led to efficient harmonics generation in such plasma formations [23,24,25,26]. The harmonics started from H_11_ and extended up to H_29_. Notice that the ablated Au 10 nm NPs on paper also demonstrated the harmonic range between H_7_ and H_29_, however, the intensities of H_11_, H_13_, H_15_ and H_17_ from the plasmas produced on the Au 10 nm NPs on paper were 40, three, four and five times stronger with regard to those from the ablated paper. The significant enhancement of HHG efficiency from Au NPs on paper is attributed to the influence of the surface plasmon resonance of Au NPs which causes stronger absorption of incident laser light resulting in larger amount of Au during propagation of the driving pulses compared to other samples.

This finding is supported by the experimental measurements of the complex dielectric function of gold [27]. Localized surface plasmon resonance increases the local electrical field around metal nanoparticle, which can in principle lead to effective decreasing of ionization potential. According to three-step model of HHG this decreased ionization potential results in higher conversion efficiency for the low-order part of HHG plateau and at the same time causes the shortening of the cutoff and plateau range. Additionally, local field enhancement is attributed to the collective motion of free electrons confined in narrowly localized regions, similar to that observed in colloidal nanoparticles exposed to an external electromagnetic field.

A method of HHG allowed exploiting the local field enhancement induced by plasmons within a metallic nanostructure consisting of bow-tie-shaped gold elements on a sapphire substrate [28,29]. HHG resulting from the illumination of plasmonic nanostructures with a short laser pulse of long wavelength was also studied in [30]. It was demonstrated that both the confinement of electron motion and the inhomogeneous character of laser electric field play an important role in the HHG process and lead to a significant increase of the harmonic cutoff. Field enhancement of plasmon nanoparticles deposited on substrate leads to enhanced second and third harmonics generation, as well as higher-order harmonics, which was reported in several works [31,32,33,34,35,36,37,38].

In our case, the harmonic intensity obtained from ablated Au 10 nm NPs on paper was approximately three and 14 times stronger with regard to Au NPs on glass and Au bulk, respectively. We estimated the conversion efficiency of samples using the comparison with known results from other plasma. The conversion efficiency from previous measurements of harmonic generation in the plasmas produced on the surface of bulk Ag was reported to be 8 × 10^−6^ [39]. In the case of Au bulk plasma at similar conditions, the HHG conversion efficiency was almost ¼ with regard to ablated bulk silver. Hence the conversion efficiency of in Au plasma was estimated to be 2 × 10^−6^. Therefore, the HHG conversion efficiency in the plasma produced on the Au NPs glued on paper was determined to be 3 × 10^−5^.

### 3.2. Role of Different Parameters of Driving and Heating Pulses on the HHG Efficiency in Au NP Containing Plasmas

The sample’s absorption and evaporation show insignificant wavelength dependences of employed 1064 nm (ns pulses) and 800 nm (ps and fs pulses) laser sources due to closeness of their wavelengths. Meanwhile, pulse duration strongly influences the ablation and harmonic emission due to different time scales of interaction with samples.

**Figure 4:**
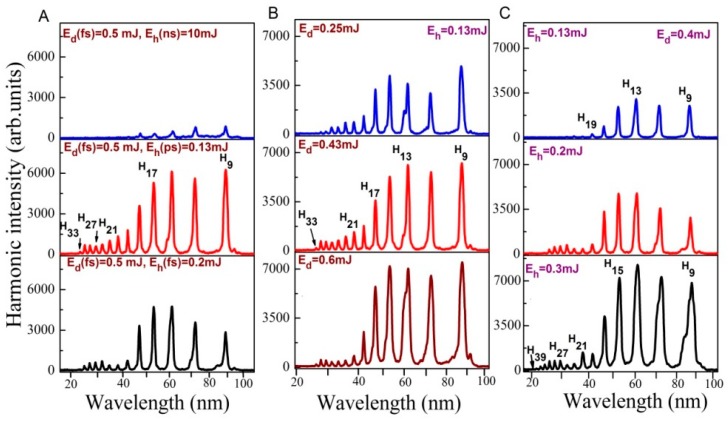
Harmonics spectra from ablated bulk Au at (**A**) different energies of ns heating pulses, (**B**) different energies of fs driving pulses, and (**C**) different energies of ps heating pulses.

The effect of heating pulse duration on HHG from ablated Au bulk target is presented in Figure 4A. Maximum harmonic intensity for ablated Au bulk target using ns heating pulses was observed at *E*(ns) = 10 mJ with harmonics extended up to H_21_ (upper panel). Harmonic yield decreased at *E*(ns) > 10 mJ due to the growth of free electrons density and phase mismatch between interacting waves. In the case of ps and fs heating pulses (two bottom panels of Figure 4A), harmonics extended up to H_33_. The intensities of harmonics in the case of heating ps and fs pulses were 5 and 4 times larger compared to the ns pulses induced ablation, while the driving pulse and heating pulse fluencies were smaller. This variation of HHG yield confirms the fact that heating pulse duration affects the harmonic yield and cut off. Our studies confirm that LPP from bulk Au allows generation of strong harmonics using short heating pulses. Laser ablation using ps and fs pulses allows the formation of relatively dense plasma, with regard to ns heating pulses while electron concentration was maintained at ~10% of the plasma concentration. To achieve a similar concentration of plasma with nanosecond heating pulse, one has to use stronger fluence, which causes the appearance of a notably larger number of free electrons. These electrons significantly suppress the conversion efficiency of harmonics [40].

At similar fluence, (i) concentration of free electrons is more in laser plasma created by ns pulses with regard to fs and ps heating pulses, and (ii) the density characteristics of plasma (i.e., concentration of neutrals and single charged atoms) are high at fs and ps ablation with regard to the ablation using ns pulses. High concentration of generated free electrons leads to self-defocusing and self-modulation of the driving laser pulses resulting in the phase mismatch between the driving and harmonic waves. Hence, the possibility of phase-mismatch using ns heating pulses is larger with regard to ps and fs heating pulses. Therefore, the efficient generation of highest harmonics from the Au plasma produced by fs and ps heating pulse is attributed to better phase matching conditions between the driving pulses and harmonic waves compared with the ns-induced LPP.

Figure 4B,C shows the variable harmonic intensities with respect to variation in the energies of heating and driving laser pulses. The harmonic intensity and harmonic range were increased with the growth in driving and heating pulse fluencies. These results illustrate that harmonics intensity and cutoff produced from the plasma of Au bulk target strongly depend on the driving and heating pulse energies.

With increase of heating pulse and driving pulse energies, the particle density and number of photons available for particle acceleration increase leading to the growth of harmonic yield and extension of harmonic cutoff. The decrease in harmonic efficiency after crossing certain energy of heating pulses is due to the growth of free electrons density, which leads to the self-defocusing and self-modulation of fs pulse resulting in phase mismatch [41,42,43].

The diameter (2*w*_0_) of the focused radiation was 64 µm. The corresponding Rayleigh length was *z_o_* = *k*(*w*_0_)^2^/2 = 4 mm. Here, *k* is the wave number and *w*_0_ is the beamwaist radius. For the plasma length of 0.3 mm, the driving beam was interacted with the plasma plume at the conditions of the plain wave propagation. The coherence length for mid-term harmonic (*q* = 21) (*L*_coh_(mm) ≈ 1.4 × 10^18^(*q* × *N*_e_)^−1^ [44]) was equal to 2 mm at the concentration of free electrons *N*_e_ = 3 × 10^16^ cm^−1^ corresponding to 10% of plasma concentration (3 × 10^17^ cm^−1^). At these conditions, no phase mismatch occurs, since the coherence length is larger than the size of nonlinear medium. The over-excitation of gold target causes the growth of free electrons concentration until the conditions when almost all particles became ionized. In that case, the coherence length decreases down to 0.2 mm, which caused the strong phase-mismatch between interacting waves inside the 0.3 mm long medium.

**Figure 5:**
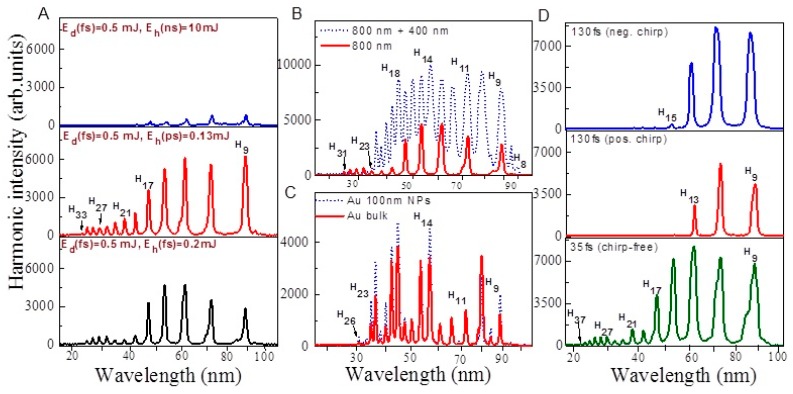
Harmonics spectra from the plasma of bulk Au. (**A**) single-color driving pulses (800 nm) and different pulse durations of heating pulses, (**B**) single- and two-color (800 + 400 nm) driving pulses while using 0.2 mJ femtosecond heating pulses, (**C**) TCP of ablated bulk Au and Au 100 nm NPs, and (**D**) application of chirped laser pulses.

Figure 5A shows the harmonic spectra obtained from ablated Au bulk target using SCP of LPP and different heating laser pulse duration. The obtained harmonics were extended up to H_33_ in the case of shortest heating pulse duration. The harmonic cut off was decreased in the case of TCP (H_23_, Figure 5B) as compared to SCP (H_31_) due to application of shorter wavelength component of pumping radiation. The harmonic intensities obtained using TCP were two times stronger with regard to SCP (Figure 5B), despite of small conversion efficiency of second-harmonic radiation in BBO (~2.5%) and small ratio of 800 and 400 nm energies (1:40). It was suggested that TCP generates stronger harmonics because of the formation of quasi-linear field, selection of a short quantum path component, which has a denser electron wave packet, and higher ionization rate compared with SCP [45]. TCP generated broader harmonics with regard to SCP due to self-modulation of the driving pulses in the plasma area (Figure 5B). Figure 5C shows the harmonic spectra obtained from ablated Au bulk and Au 100 nm NPs using TCP geometry and ns heating pulses. One can see that ablated Au 100 nm NPs produced stronger harmonics as compared to ablated Au bulk target in two-color configuration as well.

Figure 5D shows the effect of laser chirp on the generation efficiency of harmonics. The reduced harmonic cutoff in the case of chirped laser pulses is attributed to the reduced intensity at longer pulse duration. The harmonics were insignificantly red- and blue-shifted in the case of positively and negatively chirped 130 fs laser pulses. The variation of the sign of laser chirp also affected the conversion efficiency of harmonics. The low-order harmonics (e.g., H_9_ and H_11_) were larger in the case of negatively chirped 130 fs pulses with regard to positively chirped pulses. The neutrals and ions presented in plasma can be responsible for additional chirp-induced blue- and red-shifts. Particularly, self-phase modulation of laser pulses during propagation of the leading part of laser pulse through the plasma containing neutral and ions might be the reason for observed red-shift of harmonics. Previously, similar scenario has been observed in Xe gas jet and LPP [46,47].

### 3.3. Numerical Simulations of Au Nanoparticles Formation During Laser Ablation

**Figure 6:**
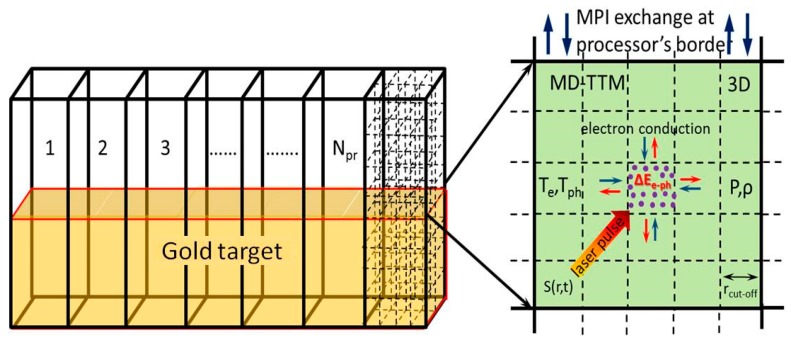
Computational box for simulations of laser-induced formation of Au NPs during the laser ablation process on the experimental scale [52]. The box is divided to the number of subcells N_x_ and N_y_ in X and Y directions correspondingly to be processed by N_x_*N_y_ processor cores (left) in multiprocessing regime with the MPI library. Each core is divided to 3D mesh and shown in 2D view (right) with an illustration of the MD-TTM model scheme [51]. There we consider the effect of free carriers via the electron, T_e_, and phonon, T_ph_ temperatures dynamics to describe the laser light absorption, S(r,t), the process of thermal energy exchange between electrons and phonons, ΔE_e-ph_, and fast electron heat conduction.

In order to support the experimental data and investigate the mechanism of NPs formation at different irradiation conditions, we performed series of MD-based modeling [48]. The classical Molecular Dynamics method can describe the kinetics of fast non-equilibrium phase transition processes at atomic precision. This method, however, does not have free electrons included explicitly and, therefore, cannot address the processes of laser light absorption, laser-induced electron-phonon non-equilibrium, and fast electron heat conduction. The last three phenomena are playing a determinant role during short laser pulse interactions with metals [49] and can be described within the frames of the Two Temperature Model (TTM) [50], where the effect of free carriers is account for via the electron-phonon temperature dynamics. In the hybrid MD-TTM method we combine the advantages of both MD and TTM methods so that all the above mentioned processes are described with the scale of a single computational approach. The basics of our atomistic-continuum MD-TTM approach are described in [51]. The schematic view of the total computational cell for the simulation of Au NPs formation in vacuum is shown in Figure 6. The spot size of the laser beam focused on the metal surface is considered large enough (0.3 mm) as compared to the lateral size of the modeling cell. The lateral size of the computational box also suits the requirements on the identical comparison of the simulation results with the experiment data.

Based on above assumptions, the total supercell consisting of ~90,000,000 atoms was taken with dimensions of 65 × 65 × 400 nm in X, Y, and Z directions respectively. While Periodic Boundary conditions were imposed in X and Y directions, to avoid unnecessary and expensive MD integrations in deep bulk of the material we imposed the Non Reflective Boundary conditions in Z at the depth of 400 nm from the metal surface. The total computational box was divided by number of cells and each of them was processed by an individual processor core. At the same time, the applied model was solved in 3D mesh inside of each processor cell (Figure 6). A similar approach for the modeling of the laser-induced processes on the experimental scale is described in our previous study on ultrashort laser pulse nanostructuring processes [52].

For the direct comparison of our simulation results with the experiments, our atomistic-continuum approach implemented a realistic interatomic potential for Au [53]. For an equilibrium crystal at P = 0 GPa and T = 0 K the potential gives −367.609 kJ/mole for the cohesive energy, 179.4 GPa for the bulk modulus, and 0.4065 nm for the lattice constant. Furthermore, this potential represents the experimental thermophysical properties of the modeled material (such as equilibrium melting temperature, heat capacity, volume of melting, and linear thermal expansion coefficient) with an accuracy of more than 99.5%. For instance, such an important property in our model as the melting temperature T_m_ = 1343 K was computed in a series of liquid–crystal coexistence simulations and showed a good agreement with its experimental value of T_exp_ = 1337 K. The relation between the incident and the absorbed fluencies is based on the reflectivity function of gold and is taken from the tabulated values of extinction coefficients [54] for the given wavelengths of the used laser pulses (800 and 1064 nm). In order to investigate the target’s response to a laser pulse at different regimes, we perform three simulations for the pulse durations of 300 fs, 4 ps, and 100 ps that are correspondingly much shorter, comparable, and much longer than the characteristic electron-phonon equilibration time *τ_e-ph_* in gold (~10 ps [55]).

**Figure 7:**
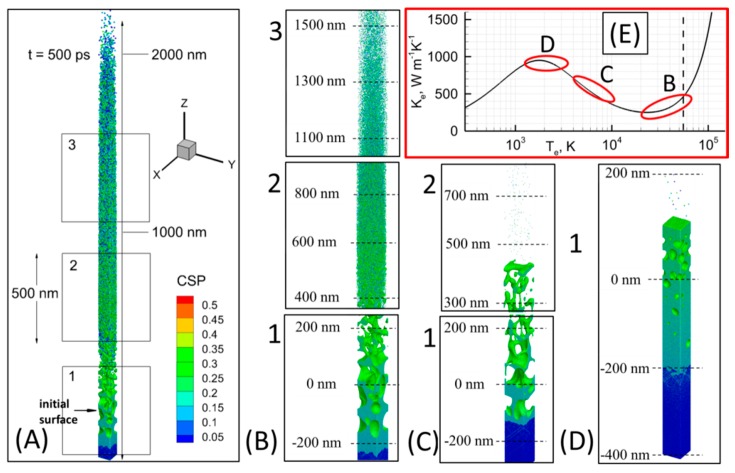
The atomic snapshots are shown for a general view on the ablation plume formed in vacuum. (**A**) at the time of 500 ps. The three zones of the NPs formations are shown for the case of 0.3 ps pulse (λ = 800 nm, F_inc_ = 2 J cm^−2^) in (**B**). Two zones of NPs formations are shown for the case of 4.0 ps pulse (λ = 800 nm, F_inc_ = 2 J cm^−2^) in (**C**). One zone of the material foaming process is seen for the case of 100 ps laser pulse (λ = 1064 nm, F_inc_ = 10 J cm^−2^) in (**D**). The atoms are colored by CSP for identification of the local crystal structures as follow: solid < 0.08 < defects < 0.12 < liquid < 0.25 < surface < 0.50 < vapor. The free (volatile) particles are blanked here for a better visualization of the NPs formation process. The electron heat conductivity *k_e_* as a function of electronic temperature *T_e_* is shown in the red rectangle (**E**) with red ovals correspondingly indicating the characteristic conductivity values for all three pulses (B) 0.3 ps, (c) 4.0ps, and (d) 100ps.

The results of modeling of NPs formation due to a laser pulse focused on a thick Au target can be seen in Figure 7 for three pulse durations. In Figure 7A one can see a general view of the ablation plume caught at the time of 500 ps due to a 300 fs laser pulse (*λ* = 800 nm) ablation at the incident fluence of 2 J cm^−2^. Here we can clearly identify three zones: “1”, “2”, and “3”, where NPs form with different size and shape. These three zones are zoomed for a more detailed observation in Figure 7B, where the atoms are colored by Central Symmetry Parameter (CSP) for identification of their local structure: solid < 0.08 < defects < 0.12 < liquid < 0.25 < surface < 0.50 < vapors. The top zone “3” contains the smallest NPs with rather spherical shape and the characteristic size of ~2–3 nm. The zone “2” has the particles of rougher form, but of bigger size (~5–15 nm). And finally, the area “1” shows the part of the material, where the formation of NPs is still in progress due to spallation mechanism, with the forming clusters of ~30–40 nm characteristic size.

For the case of 0.3 ps pulse duration (Figure 7B) the rate of target heating is determined by the characteristic time of the electron-phonon relaxation process, which is on the order of 20ps [55]. Providing the efficient laser heating depth large enough (~200 nm), the mechanical relaxation rate is weaker and the high compressive stresses are built up in the vicinity of the material surface [56]. These conditions are frequently referred to as the regime of internal stress confinement, which provides the mechanical damage of the target with the subsequent formation of large NPs. However, due to the relatively large values of the electronic temperature developed during the pulse (up to 45,000 K), the conductivity values (*k_e_*) of the excited free carriers is dynamically changing from *k_e_* ~ *T_i_*/*T_e_* dependence when *T_e_* is low to *k_e_* ~ *T_e_*/(*T_i_* + *T_e_*^2^) dependence for higher values of *T_e_*. This situation leads to accumulation of the laser deposited energy in the proximity of the surface and referred to as thermal confinement regime [56], is indicated in Figure 7E in the red rectangle, where we plot the conductivity of free electrons in gold as a function of electronic temperature at the fixed value of the lattice temperature, and marked as *B*.

While simulating the process of NPs formation with MD-TTM model, we can monitor all thermophysical properties of matter (pressure, density, temperature) and also density and temperature of the electrons (due to the TTM part of the combined MD-TTM model). Therefore, the electron temperature at any moment of simulation time is known as well, and its maximum value during the 0.3 ps laser pulse interaction with thick Au sample was measured during the simulation (not shown explicitly, but its characteristic values are indicated in the insertion of Figure 7 as ovals for each of the simulation case). Note that thermal confinement regime results in the lattice temperature at the surface reaching its critical value (~7000 K) and the explosive boiling process serves as the driving mechanism for the material ejection. The whole ablation process for the case of 0.3 ps pulse, therefore, is a mixture of the thermal damage at the surface (i.e., ablation, area “3” in Figure 7B), and the mechanical damage of deeper parts of the material (i.e., spallation, area “1” in Figure 7B).

The material ejection process due to the explosive boiling mechanism results in a high velocity of the small NPs, shown in the region “3” Figure 7B, where they move with the speed of ~6500 m s^−1^. At the same time, the intensive evaporation process efficiently cools down the material’s surface due to the transfer of thermal energy to the enthalpy of vaporization. Thus, the NPs with the lower kinetic energy, but with large size are shown in the region “2”, where they move at a slower speed of ~3500 m s^−1^. Finally, when the material’s surface loses most of its thermal energy, we observe the ordinary material ejection process due to the spallation mechanism forming therefore the region “1” and moving roughly at the speed of ~750 m s^−1^. Further evolution of this ablation plume will result in a more pronounced segregation of the generated NPs in accordance with their size and the lift-off velocity.

The result of 4.0 ps laser pulse interaction with gold at the incident fluence of F_inc_ = 2 J cm^−2^ (λ = 800 nm) is shown in Figure 7C. Unlike the previous situation, here we observe the formation of large droplets of the material without noticeable contribution due to small clusters and free atoms shown in zone “2”. The process of foam formation due to spallation is seen in zone “1”. This time, since the pulse is longer, the laser intensity at the peak of the pulse is much lower as compared to the case of 0.3 ps pulse. The elevation of the electronic temperature therefore, is just 15,000 K, which corresponds to the scaling of electron conductivity function as *k_e_* ~ *T_e_*/*T_i_*, and reflected in Figure 7E in the red rectangle by the marker *C*. Therefore, the deposited laser energy will efficiently penetrate the bulk of the material resulting in the establishment of temperature and pressure gradients by the time of the electron-phonon equilibrium on a spatial scale of ~200 nm.

The result of 100 ps laser pulse interaction with gold at the incident fluence of F_inc_ = 10 J cm^−2^ is shown in Figure 7D, where the only foaming process in zone “1” is observed. The 100 ps laser pulse, being much longer than the characteristic electron-phonon relaxation time, cannot induce strong electron-phonon non-equilibrium conditions. The electronic temperature therefore, does not reach high values and limited roughly within 5000 K, where the laser deposited energy dissipation channel through the electron heat conduction is strong, but the induced lattice heating is weak. This situation is indicated in Figure 7E in the red oval marked *D*.

From the performed simulation we can thus conclude that, due to a specific dependence of the electron heat conductivity function of gold from the induced electronic temperature, the shorter (fs) laser pulses or the pulses at sufficiently high incident energy can result in explosive boiling mechanism of the material removal process and NPs generation of few nm in size and of high yield. This corresponds to the experimental data above and can be related to the measurements presented in Figure 4.

**Figure 8:**
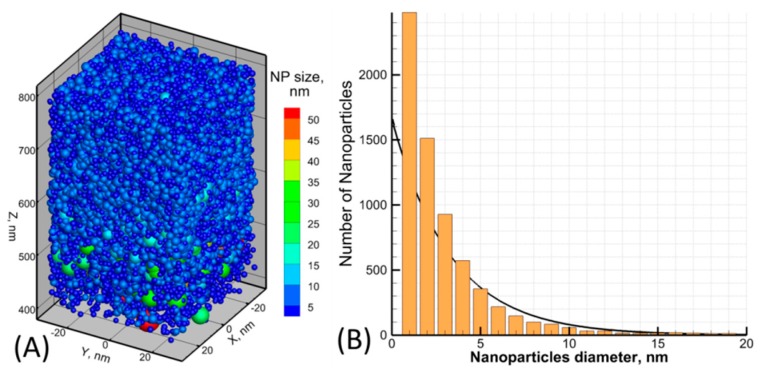
The NPs formed due to 0.3 ps laser pulse interaction with a thick Au target are shown for the area “2” in Figure 7B between 400 nm and 800 nm of the ablation plume (**A**). The NPs are colored and sized in accordance to their diameter value. The NPs size distribution for the same conditions is shown in (**B**). The exponential fit is applied for estimation of the NPs mean size of ~3–4 nm.

As it was pointed out above, the evolution of the ablation plume is governed by the size and the speed of the expelled NPs. Therefore, depending on the delay between the driving and the probe pulses, one can succeed in HHG process due to the NPs of different size. In Figure 8A we perform a visualization of the NPs size and their position in the ablation plume between 400 nm and 800 nm from the position of the initial surface. The visual analysis allows concluding that, apart from the number of monoclusters (consisting of few tens of atoms), a significant input to the efficiency of HHG process will be given due to NPs of ~5 nm. A more careful analysis of the same ablation plume volume allows extracting the mean size of the NPs, ~3–4 nm, from their size distribution, shown in Figure 8B. This supports the experimental measurements of the deposited NPs size of 5 nm for these conditions and can also indicate the use of the probe pulse delay (or the distance of the probe pulse generation from the initial surface) for manipulation with the efficiency of the HHG process that was also confirmed experimentally.

To compare the simulations and experimental data and to prove the presence of clusters in gold-ablated plasmas under optimal conditions of harmonic generation, the morphology of the deposited debris from Au plasma created during target surface ablation by laser pulses was analyzed. Laser ablation of a solid material is a widely accepted technique for the generation of nanoparticles. However, this process has previously been studied without taking into account the role of free electrons and highly excited ions, which violates the optimal conditions for phase-matched HHG.

**Figure 9:**
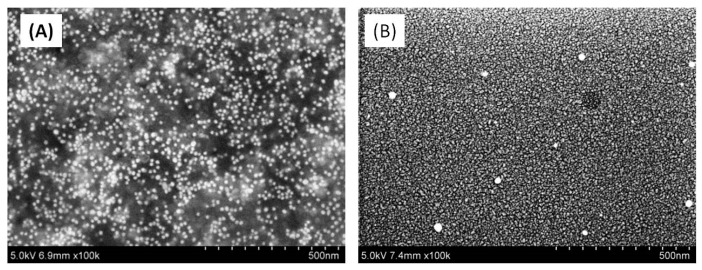
SEM images of (**A**) deposited 10 nm NPs after ablation, and (**B**) deposited NPs synthesized during ablation of Au bulk target by 200 ps pulses.

SEM measurements of the deposited debris of 10 nm Au NPs (Figure 9A) were carried out under laser ablation conditions corresponding to the optimal plasma formation for highest yield of harmonics. The substrates (glass plates and silicon wafers) used to collect the deposited material were placed at a distance of 40 mm in front of the ablation area, and the debris was further analyzed by SEM. We determined that the mean size of deposited NPs (12 nm) was close to the NPs sizes measured prior ablation (8–15 nm).

During ablation of bulk target, under weak gold plasma formation conditions, the SEM images did not reveal the presence of nanoparticles in deposited debris with sizes above the microscope detection limit (3 nm). This was probably due to the small fluence (0.5 J cm^−2^) of the heating 200 ps pulses on the target surface. Another pattern was observed upon ablation of the target using higher heating fluence (2 J cm^−2^), which caused the appearance of small nanoparticles deposited onto a nearby substrate. At these conditions, the NPs appeared in the SEM images of the deposits, with their mean size of ~5 nm, while some larger NPs of a mean size of about 20 nm and higher were also seen (Figure 9B). One has to reiterate that these characteristics of debris were measured once obtaining them at the maximum HHG conversion efficiency. These morphological studies confirmed the presence of a large number of tiny NPs and small amount of large NPs simultaneously deposited on the substrates under conditions of ‘optimal’ laser ablation. This observation points out the presence of synthesized NPs at the moment of femtosecond pulses propagation.

The concentration of these small NPs increased with the growth of heating pulse intensity. Production of nanoparticles by laser ablation of metallic targets is a well-studied phenomenon. However, the use of high ablation fluencies allowing the synthesis of large amount of Au NPs resulted in the growth of free-electron concentration, which is a most detrimental factor in HHG due to the contribution to the phase mismatch between the driving and harmonic waves. This explains why, under ablation conditions leading to NP formation in the plume of bulk metallic targets, the HHG signals are weaker with regard to the ablation of already existing Au NPs on the target surface. In that case, the presence of NPs in the plasma does not compensate for the deteriorated phase mismatch conditions caused by over-ionization and presence of large number of electrons.

## 4. Discussion

The characterization of the gold nanoparticles being presented in LPP is an important component of morphology and HHG studies, though it is very difficult to properly determine some of those parameters. Below we address a few issues related with our experiments using Au NPs and try to answer on some questions regarding these studies.

(1) Can the laser radiation destroy or modify Au NPs? Yes, it can. NPs can be disintegrated by the radiation of the heating pulses. We analyzed the debris of deposited Au NPs under the irradiation by ns, ps, and fs pulses. The mean size of NP debris was dominantly the same as of the initial NPs (10 and 100 nm), though the presence of the smaller-sized wing in the histogram of size distribution pointed out the appearance of disintegrated NPs. Their ratio was insignificant, since we ablated Au NPs at the fluencies allowing evaporation of those particles without the notable modification of their structure. Stronger irradiation of targets led to the appearance of large aggregates and small NPs in the nearby substrates, alongside with the decrease of HHG conversion efficiency attributed to large concentration of free electrons.

(2) How much of them remained from pulse to pulse? As for the remained particles on the target surface, the concentration of them did not change, since only insignificant part of NPs was ejected from the surface during a single shot. The problem of crater formation was resolved by moving the target surface either by dragging the sample up and down or by using the rotating targets or by both above methods.

(3) How many particles escaped into the plasma? To properly analyze and determine the amount of NPs ablated and appeared in the plasma area one has to carry out the accurate measurements of the weight of NP powder before and after ablation by large number of pulses and then calculate how many particles escaped into the plasma during a single shot. Even this information is insufficient for determination of the amount of NPs participating in HHG during the interaction with fs driving pulses, since the plasma cloud had relatively large sizes (a few mm, in accordance with the observations of the emission of incoherent radiation of the plasma moving out from the surface) and only small portion of them met the fs driving beam. How much NPs were inside the “tube” with the diameter ~64 µm (i.e., sizes of the focused driving beam) and length ~0.4 mm (diameter of spreading plasma at the distance of ~0.5 mm from the target surface) at the moment when largest amount of them reaches the axis of propagation of the fs pulse remains unknown.

(4) What was their concentration? Here we can talk only about the averaged concentration of NPs in the plasma cloud at the moment of propagation of the driving fs pulses. Earlier, estimates and calculations using code HYADES of the density of ablated particles at the conditions suitable for efficient HHG reported on the 2 × 10^17^ cm^−3^ concentrations of silver atoms and ions in the case of efficient HHG in Ag plasma [57]. Present studies were carried out at other experimental conditions (i.e., easier ablation and lower laser heating fluencies, formation of NP cloud, presence of different groups of NPs, etc.). It is also difficult to compare the concentrations of the homogeneous plasma containing separated atomic and ionic species and of the plasma containing ultrasmall “solid” species like NPs.

From the CSP values of the atoms in Figure 7 one can easily see that the density of the formed NPs is close to the density of solid. Furthermore, while modeling of the NPs formation process with the MD-TTM model, it is easily seen in from numerical analysis that the formation of the NPs takes place via the establishing of metallic bonding between the atoms, that only possible (via the interatomic function construction) if the atomic density is close (within 10%) to that of the solid.

An explanation for intense harmonic generation from Au NPs could be the higher concentration of neutral atoms due to the presence of nanoparticles. Unlike single atoms and ions, whose density quickly decreases due to plasma expansion, the NPs retain densities that are close to its solid state, while distance between NPs in plasma jet is notably larger as compared with the distance between particles in atoms-containing LPPs. Concentration of atoms in solid species depends on its density and varies in the range of 10^22^–10^23^ cm^−3^. Combined with the higher harmonic efficiency of neutral atoms compared with their ions, the neutral atoms within the NPs could generate high-order harmonics efficiently.

How many atoms in the nanoparticle become optimal for efficient generation of coherent extreme ultraviolet radiation using a whole ensemble of particles, which allow increasing the number of photons of high-order harmonics, remains a puzzle despite the fact that to date numerous experiments using the ablated NPs were conducted [58,59,60]. Qualitative assessments predict that presence of the particles containing a few hundred to a few thousand atoms in the area of interaction with strong laser field may lead to the maximal growth of generated harmonics. The comparative analysis of HHG spectra generated in the plasmas excited on the surfaces of pure paper and Au NPs-contained paper show the advantages of ablated NPs-containing targets and influence of these species to the enhancement of harmonic yield at the same conditions of experiment.

There are still a lot of issues that have to be clarified in the case of application of the used Au NPs for HHG. It remains a puzzle how the composition and spatial scales of the NPs affect the HHG efficiency and the cutoff frequency. There remain also other questions regarding the charge state of this plasma medium, which could be resolved only by using time-of-flight mass spectrometry. Particularly, are the Au NPs were charged before the driving pulse comes? How many electrons are ionized under the high intensity pump pulse from each NP? What are the exact ionization potentials of the chosen Au NPs and how do they affect the HHG cutoff? Neither of those questions still found the proper answer. It is difficult to resolve numerous issues while dealing with NPs in plasmas due to availability of only indirect methods of the measurements of plasma parameters. Some additional abovementioned issues also still wait to be resolved.

Authors should discuss the results and how they can be interpreted in perspective of previous studies and of the working hypotheses. The findings and their implications should be discussed in the broadest context possible. Future research directions may also be highlighted.

## 5. Conclusions

The 5- and 40-fold enhancement of harmonic yield was obtained from ablated Au NPs on paper as compared with ablated Au NPs on glass and Au bulk target. The harmonic cutoffs obtained from ablated Au NPs on paper, Au NPs on glass and Au bulk target were 29th, 21st and 21st orders, respectively, in the case of nanosecond heating pulses. In addition, effects of heating pulse duration, TCP and laser chirp on harmonic yield were studied in Au NP-contained LPPs. Enhanced harmonic intensity was obtained from ablated Au bulk target by decreasing the heating pulse duration. The application of picosecond and femtosecond heating pulses for plasma formation allowed generation of 5 and 4 times stronger harmonics with regard to the nanosecond heating pulses. The harmonic cutoffs were 33rd and 39th orders in the case of picosecond and femtosecond heating pulses, respectively. The enhanced harmonic intensity was demonstrated from ablated Au bulk target by applying TCP (800 nm + 400 nm) of LPP. Two-fold enhancement of harmonics was observed using TCP of ablated Au bulk target with regard to SCP. Negatively chirped 130 fs pulses enhanced 9th and 11th harmonics with regard to chirp-free (35 fs) and positively chirped 130 fs pulses. Performed MD-based simulations of NPs formation at different pulse durations and the incident fluencies supported the experimental measurements and revealed the main driving mechanisms responsible for the size and morphology of the generated particles.
